# Cancer Susceptibility Candidate 9 (CASC9) Promotes Colorectal Cancer Carcinogenesis via mTOR-Dependent Autophagy and Epithelial–Mesenchymal Transition Pathways

**DOI:** 10.3389/fmolb.2021.627022

**Published:** 2021-05-04

**Authors:** Md Zahirul Islam Khan, Helen Ka Wai Law

**Affiliations:** Department of Health Technology and Informatics, Faculty of Health and Social Sciences, The Hong Kong Polytechnic University, Kowloon, Hong Kong

**Keywords:** cancer susceptibility candidate 9, long non-coding RNA, colorectal cancer, autophagy, epithelial-mesenchymal transition

## Abstract

**Background:**

Colorectal cancer (CRC) is the third most common cancer worldwide. Many recent studies have demonstrated that different long non-coding RNAs (lncRNAs) are involved in the initiation, advancement, and metastasis of many cancers including CRC. Cancer susceptibility candidate 9 (CASC9) is an lncRNA that has been reported in many cancers, but its role in CRC is poorly understood. In this study, we aimed to examine the expression of CASC9 in CRC cell lines and to determine the mechanism of action of CASC9 in CRC carcinogenesis.

**Methods:**

The expression of CASC9 in CRC tissues was compared with normal samples from publicly available datasets in The Cancer Genome Atlas (TCGA) and The Encyclopedia of RNA Interactomes (ENCORI). CASC9 expression was further verified in four CRC cell lines (DLD1, HT-29, SW480, and HCT-116) and normal colorectal cell line (CCD-112CoN) by real-time quantitative polymerase chain reaction (RT-qPCR). After gene silencing in HCT-116 and SW480, Cell Counting Kit-8 assay, clonogenic assay, and wound healing assay were performed to evaluate cell proliferation, viability, and migration index of cells. Western blotting was used to explore the key pathways involved.

**Results:**

CASC9 was significantly upregulated as analyzed from both public datasets TCGA and ENCORI where its overexpression was associated with poor survival of CRC patients. Similarly, CASC9 was significantly overexpressed in the CRC cell lines compared with normal cells studied. The silencing of CASC9 in HCT-116 and SW480 attenuated cell proliferation and migration significantly. Furthermore, pathways investigations showed that silencing of CASC9 significantly induced autophagy, promoted AMP-activated protein kinase (AMPK) phosphorylation, inhibited mTOR and AKT signaling pathways, and altered epithelial–mesenchymal transition (EMT) marker protein expression.

**Conclusion:**

We demonstrated that silencing of CASC9 contributes to the reduced CRC cell proliferation and migration by regulating autophagy and AKT/mTOR/EMT signaling. Therefore, CASC9 plays an important role in carcinogenesis, and its expression may act as a prognostic biomarker and a potential therapeutic target of CRC management.

## Introduction

Colorectal cancer (CRC) is the third most commonly diagnosed malignancy worldwide (Bray et al., 2018; Rawla et al., 2019). Statistics revealed that in 2018, nearly 1.8 million new CRC cases were reported with ∼0.9 million CRC deaths worldwide (Araghi et al., 2019). In the past decade, CRC treatment has progressed remarkably, but late diagnosis and development of metastasis are the main obstacles leading to failure in CRC treatments (Islam Khan et al., 2019; Liu et al., 2020). Therefore, it is important to identify novel targets for early diagnosis and design new therapy to minimize the CRC mortality globally.

Long non-coding RNAs (lncRNAs) are fragments of RNA that lack protein coding transcript. They are members of non-coding RNAs (ncRNAs). More specifically, lncRNAs contain more than 200 nucleotides and are routinely transcribed by RNA polymerase-II in the human genome (Zampetaki et al., 2018; Islam Khan et al., 2019). In recent years, accumulating evidence suggested that lncRNAs sometimes behaved like regulatory molecules to control gene expressions. They are involved in the signaling pathways responsible for cell growth, development, and metabolic processes (Lin and He, 2017; Sparber et al., 2019). In cancer, lncRNAs are associated with each stage of tumor initiation, progression, and poor prognosis by enabling drug resistance (Galamb et al., 2019; Qi et al., 2020). The aberrant expression of lncRNAs alters the major oncogenic signaling cascades, for example, WNT/B-catenin, P53, mTOR, PI3K/Akt, AMP-activated protein kinase (AMPK), EGFR, NOTCH, and MAPK pathways (Kessler et al., 2013; Khan et al., 2015; Sever and Brugge, 2015).

In the past decade, many investigators concluded that abnormal expression of lncRNAs may be responsible for CRC inception, progression, and poor treatment outcomes of patients (Esmaeili et al., 2020; Qi et al., 2020). For instance, UNC5B antisense lncRNA 1 (UNC5B-AS1) has been shown to reduce apoptosis, accelerate CRC progression, and result in metastasis (Zhang Y. et al., 2020). Liu et al. (2020) illustrated that higher expression of KCNQ1OT1 promotes CRC carcinogenesis by enhancing aerobic glycolysis and stabilization of hexokinase 2 gene (Chen C. et al., 2020). Likewise, FOXC2-AS1 promoted CRC progression via stabilizing FOXC2 and Ca^2+^ channel–controlled FAK signaling pathway (Pan and Xie, 2020). Bin et al., 2021 demonstrated that overexpression of EPB41L4A-AS1 is associated with CRC development. It activates Rho/Rho-associated protein kinase to promote CRC cell growth, proliferation, and migration (Bin et al., 2021). Shan et al. (2016) revealed that Linc-POU3F3 acts as an oncogenic gene in CRC to promote initiation, progression, and metastasis *in vitro*. In contrast, silencing of linc-POU3F3 reduced CRC carcinogenesis by inducing autophagy-mediated apoptosis process (Shan et al., 2016). Another study showed that TTN-AS1 silencing exerts its tumor suppressor activity through the reduction of epithelial–mesenchymal transition (EMT) process and PI3K/AKT/mTOR signaling (Cui et al., 2019). Overall, this indicates that lncRNA expression may promote or suppress CRC independently by regulating diverse molecular pathways.

Cancer susceptibility candidate 9 (CASC9), a recently discovered lncRNA, consists of four transcript variants CASC9-201, CASC9-202, CASC9-203, and CASC9-204. CASC9 earned significant attention of researchers because of the potential roles of its transcript variants in association with the pathogenesis of various cancer (Sharma et al., 2020). Recently, Luo et al. (2019) revealed that upregulation of CASC9 is associated with advanced TNM stage and poor prognosis of CRC. In addition, CASC9 exerts its oncogenic activity through the phosphorylation of SMAD3 and TGF-β signaling *in vitro* (Luo et al., 2019). Another study performed by Ding et al. (2020) reported that CASC9 upregulation promotes CRC carcinogenesis by regulating miR-193a-5p and ERBB2 expression. Although these two studies demonstrated some roles of CASC9 in CRC, the molecular mechanisms of CASC9 in promoting carcinogenesis still remain largely unknown. Our present study aimed to explore the expression of CASC9 in CRC cell lines and to determine the roles of CASC9 in mTOR-dependent autophagy and EMT, which are associated with CRC progression. Our findings suggest that CASC9 might be used to evaluate CRC prognosis, and it may be used as a novel therapeutic target for CRC patients.

## Materials and Methods

### Data Mining and Analysis

The differential expressions of CASC9 in CRC and the adjacent normal tissues were obtained from two publicly available dataset: The Cancer Genome Atlas (TCGA)^1^ program under the National Cancer Institute and The Encyclopedia of RNA Interactomes (ENCORI) (Li et al., 2013). From TCGA–colon adenocarcinoma (COAD) analysis, there were 275 tumor samples compared with 349 adjacent normal tissues for the CASC9 expression profile analysis. *P* < 0.01 and fold change Log_2_FC > 2.00 were considered as cutoff values to plot CASC9 boxplot. The overall survival of CASC9 was determined by using TCGA-COAD dataset with median cutoff and 95% confidence interval, counting the number of transcript per million (TPM), and considering the hazard ratio (HR). Likewise, the CASC9 expression was extracted from the ENCORI-COAD dataset containing 471 tumor samples, and 41 normal tissues were used to generate the boxplot. The log-rank *P* < 0.05, HR, and high/low expression number were used to plot the survival curve from ENCORI-Pan-Cancer (Li et al., 2013).

### Cell Culture

Human normal colon cells, CCD-112CoN, were acquired from American Type Culture Collection (ATCC) (Manassas, VA, United States), and human CRC HT-29 cell was acquired from PerkinElmer, Inc. (Waltham, MA, United States). In addition, three more human CRC cell lines, namely, DLD-1, HCT-116, and SW480, were kindly provided by our collaborator, Prof. Jun Yu, Department of Medicine and Therapeutics, Institute of Digestive Disease, The Chinese University of Hong Kong. CCD-112CoN cells were maintained with 10% fetal bovine serum (FBS) (Gibco, United States) in Eagle minimum essential medium (ATCC), whereas HT-29, DLD-1, HCT-116, and SW480 were cultured in Dulbecco modified eagle medium (DMEM; Gibco, United States) with 10% FBS. Cell culture was maintained at 37°C in 5% CO_2_ in 100% humidity.

### Dicer-Substrate Mediated Transfection

To achieve Dicer-substrate–mediated CASC9 (Dsi-CASC9) silencing, SW480 and HCT116 cells were seeded and cultured in a six-well plate. Transfection experiment was performed when the cell density reached 60–70% confluence. A lipid-based *in vitro* transfection was carried out using Lipofectamine 2000 (Invitrogen, United States) according to the manufacturer’s protocol. TriFECTa kits were purchased from Integrated DNA Technologies (IDT, United States), which contained a Dicer-substrate negative control (DSi-NC), positive control (Dsi-HPRT-S1), transfection control (Dsi-TYE 563), and predesigned Dsi-CASC9 duplex. The duplex sequences for Dsi-CASC9 were as follows: 5′-GAGAGUCAUUGGCACUAUCAAGAAA-3′ and 3′-ACCUCUCAGUAACCGUGAUAGUUCUUU-5′. The Dsi-NC and Dsi-HPRT-S1 sequences were not provided by the manufacturer.

### Complementary DNA Synthesis and RT-qPCR

Total RNA from the colon cells was extracted using RNeasy mini kit (Qiagen, Germany) according to manufacturer guidelines. The RNA concentration was measured by NanoDrop 200 (Thermo Fisher Scientific, United States). Following standard protocol, first-strand cDNA was synthesized using Superscript II and Random Hexamer (Invitrogen, United States). Master Mix LightCycler 480 SYBR Green I (Roche, Switzerland) was used to complete the quantitative reaction using LightCycler 480 Instrument II (Roche, Switzerland). Here, GAPDH was considered as the housekeeping gene, and relative expression was calculated by 2^–ΔΔ*Ct*^ method. The following self-designed primer sequences were used in this study: GAPDH, forward: 5′-TGCCATCAATGACCCCTTC-3′ and reverse, 5′-CATCGCCCCACTTGATTTTG-3′; CASC9, forward: 5′-TTGGTCAGCCACATTCATGGT-3′ and reverse, 5′-AGTGCCAATGACTCTCCAGC-3′; HPRT1, forward: 5′-TGC TCGAGATGTGATGAAGG-3′, and reverse, 5′-TCCCCTGTT GACTGGTCATT-3′. All primers were purchased from IDT (United States).

### Cell Viability Assay

After a day of transfection, cells were trypsinized and counted by hemocytometer for seeding and performing cell proliferation assay using Cell Counting Kit-8 (CCK-8, Dojindo). Cells, 3 × 10^3^, in 100 μL of complete medium were seeded and cultured in a 96-well plate. According to CCK-8 cell proliferation assay protocol, 10 μL of CCK-8 solution was added to the well. After 3-h incubation at 37°C + 5% CO_2_, the amount of formazan that represents the number of live cells was measured at an absorbance of 450 nM using a SPECTROstar Nano Microplate Reader (BMG Labtech, Germany).

### Colony Formation Assay

The colony formation assay was performed to measure the cell proliferation potential *in vitro*. After being transfected for 24 h, 1 × 10^3^ cells were seeded and cultured for around 2 weeks in a six-well plate in triplicates. At the endpoint, the colonies were fixed with a 3:1 mixture of methanol and acetic acid. A solution of 0.5% crystal violet in methanol was used to stain and visualized the colonies. The images were taken, and the numbers of colonies were counted using the ImageJ software [National Institutes of Health (NIH)].

### Migration Assay

In the migration assay, 5 × 10^4^ cells in 70 μL DMEM with 10% FBS were carefully placed in both compartments of the Culture-Insert 2 Well (Ibidi LLC, Germany). After 24 h of cell settling, the culture inserts were gently removed to create a gap of ∼500 μm for measuring the cell migration ability. Then, each well was filled with 1.5 mL of complete medium. The photographs of the wound areas were taken using an inverted microscope (Nikon, Japan) at various time points of 0, 24, and 48 h, respectively. The migration index indicating the size of the gap was measured using the MRI Wound Healing Tool in ImageJ (NIH).

### Western Blotting

Western blotting was performed using standard, established protocol as previously published (Tam et al., 2020). Briefly, protein isolation was performed using RIPA lysis and extraction buffer (Thermo Fisher Scientific, United States) with a supplement of cOmplete ULTRA Tablets, Mini EDTA-free, Easy pack Protease Inhibitor Cocktail (Roche, Switzerland). Protein concentration was quantified using BCA Protein Assay Kit (Thermo Fisher Scientific, United States), and similar amounts of proteins were loaded and run on 8–12% sodium dodecyl sulfate–polyacrylamide gel electrophoresis at ambient temperature. Proteins were then transferred onto Immun-Blot PVDF membrane (Bio-Rad Laboratories, Inc., United States), followed by 2-h blocking in 5% bovine serum albumin (BSA) (Hyclone BSA; GE Healthcare Life Science, United States) in Tris-buffered saline with a supplement of 0.1% Tween 20. Then, the blocked membrane was incubated overnight with primary antibodies: β-actin [#8457, Cell Signaling Technology, Inc., (CST, United States)], GAPDH (#2118, CST), AKT (#9272, AKT, Phosphor-AKT (#9271, CST), AMPKα (#5832, CST), phosphor-AMPKα (#2535, CST), E-cadherin (#3195, CST), N-cadherin (#13116S, CST), LC3B (#2775, CST), mTOR (#2972, CST), Phosphor-mTOR (#2535, CST), and vimentin (#5741S, CST) at 4°C. The secondary anti-rabbit immunoglobulin G (IgG), horseradish peroxide (HRP)–linked, or anti-mouse IgG-HRP–linked (#7076, CST) antibody were added and incubated with the membrane for 2 h. Afterward, Western Lightning Plus-Electrochemiluminescence (PerkinElmer, Inc., United States) was added to the membrane to visualize protein bands in a ChemiDoc MP Imaging System (Bio-Rad Laboratories, Inc., United States). The relative protein expressions were quantified using ImageJ software (NIH) with β-actin or GAPDH as internal control.

### Statistical Analysis

The mean ± standard error of mean (SEM) of at least three or more independent experiments were included for analysis. The statistical level of the experimental data was calculated by Student *t* test or one-way analysis of variance using GraphPad Prism version 8.0 (GraphPad Software, Inc., San Diego, CA, United States). *P* < 0.05 is considered statistically significant.

## Results

### CASC9 Overexpression Correlates With Poor Survival in CRC

**FIGURE 1:**
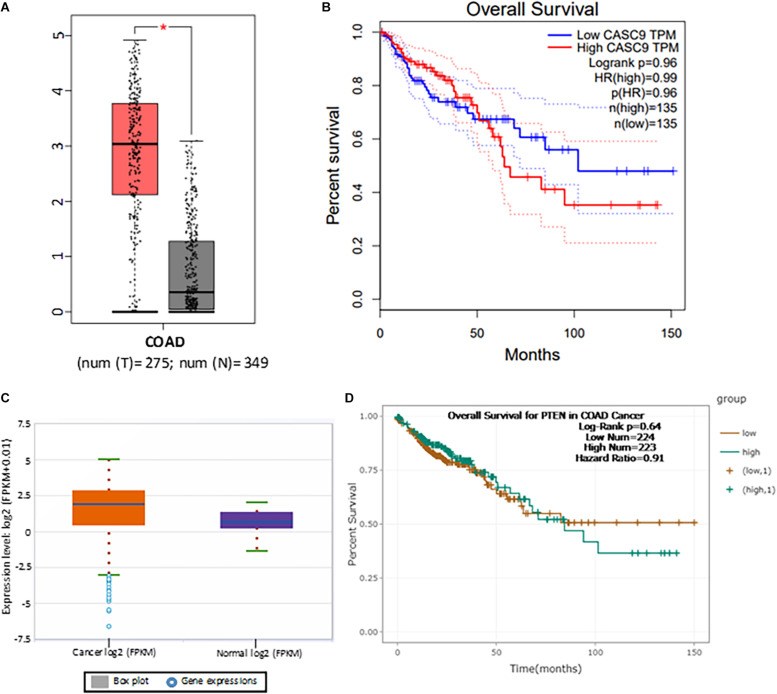
CASC9 overexpression is correlated with poor survival in CRC. **(A)** Boxplot CASC9 expressions of CRC tissues (*n* = 275) compared with normal samples (*n* = 349) from TCGA-COAD dataset (http://gepia.cancer-pku.cn/detail.php) showing that CASC9 was significantly upregulated in CRC tissues with a Log2FC cutoff value 2.0 and *P* < 0.01 (*). **(B)** The expression profiles, number of TPM, and HR (%) were used to plot overall survival. Higher expression of CASC9 in CRC tissue is associated with poor overall survival. **(C)** CASC9 expression was extracted from ENCORI-COAD dataset (Li et al., 2013). Four hundred seventeen tumor samples and 41 normal tissues were used from the dataset. Boxplot analysis showed that CASC9 was upregulated in tumor tissues. **(D)** The log-rank *P* < 0.05, HR, and high/low expression numbers were used to plot survival curve from ENCORI-Pan-Cancer. Higher expression of CASC9 reduced the overall survival of patients. TCGA, The Cancer Genome Atlas; COAD, colon adenocarcinoma; ENCORI, The Encyclopedia of RNA Interactomes; TPM, transcript per million, HR, hazard ratio.

To explore the role of CASC9 in CRC, we first searched the publicly available TCGA-COAD dataset (Figure 1A). Boxplot analysis of CASC9 showed that it was significantly upregulated in CRC samples (*n* = 275) compared with adjacent normal tissues (*n* = 349) (Figure 1A). Furthermore, we evaluated the relationship between CASC9 expression and clinical outcomes of patients. To do so, we plotted the survival curve of CRC patients according to their CASC9 expression level, number of TPM, and HR% using TCGA-COAD dataset in Gene Expression Profiling Interactive Analysis bioinformatics tool^2^. We found that the patients with higher CASC9 have poor prognosis of the disease and reduced overall survival (Figure 1B). To further confirm our findings, we explored CASC9 expression in another publicly available dataset, ENCORI-COAD (Figure 1C). CASC9 expression was found to be overexpressed in 417 CRC tumor samples compared with 41 normal tissues (Li et al., 2013). Similarly, we plotted the survival curve for CRC patients based on log-rank *P* < 0.05, HR, and high/low expression profiles of CASC9 in the dataset. Higher expression of CASC9 from ENCORI-COAD dataset showed a reduced overall survival of patients compared to normal (Figure 1D).

### CASC9 Can Be Effectively and Consistently Silenced by Dicer-Substrate siRNA Techniques in CRC Cells

**FIGURE 2:**
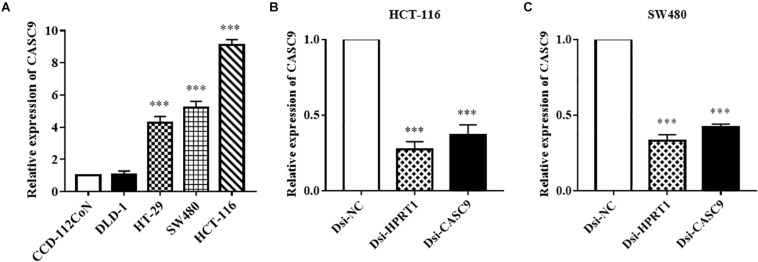
CASC9 expressions in colon cell lines and effective silencing by Dicer-substrate siRNA. **(A)** The expression of CASC9 in CRC cell lines DLD-1, HT-29, SW480, and HCT-116 was compared with normal colon cell line CCD-112CoN using RT-qPCR. The data are shown as mean ± SEM of eight independent experiments. **(B,C)** Effective and consistent silencing of CASC9 by Dicer-substrate siRNA techniques was observed. The data are shown as mean ± SEM compared to the negative control Dsi-NC. *n* = 6, ***P < 0.001.

Cancer susceptibility candidate 9 expression was measured in human CRC cell lines (DLD-1, HT-29, SW480, and HCT-116) and normal colon cell line CCD-112CoN (Figure 2A) by RT-qPCR. Similar to the observation in public datasets, the expression of CASC9 was significantly upregulated in CRC cell lines compared to normal CCD-112CoN cells (*P* < 0.001, *n* = 8). This result indicated that higher expression of CASC9 may play a role in CRC carcinogenesis. Significantly higher level of CASC9 was detected in HCT-116 > SW480 > HT-29 cells (Figure 2A). Therefore, HCT-116 and SW480 were chosen for gene silencing assay. The qRT-PCR results showed excellent knockdown efficiency of Dsi-CASC9 in HCT-116 and SW480 cells to be 63.25 ± 8.42% and 58.0 ± 6.20%, respectively (Figures 2B,C). To validate our knockdown method, gene of positive control (HPRT-1) was performed in both cell lines, and we confirmed a knockdown efficiency of more than 60% (Figures 2B,C).

### Silencing of CASC9 Reduced Cell Viability, Colony Formation, and Migratory Index of CRC Cells

**FIGURE 3:**
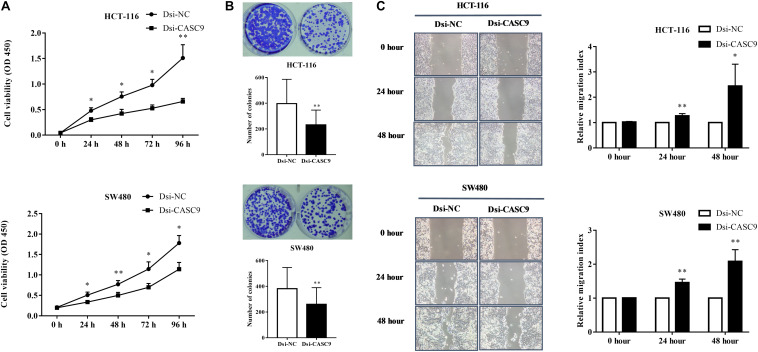
Silencing of CASC9 reduced cell viability and colony formation and increased migration index of CRC cells. **(A)** The silencing of CASC9 led to significant decrease in HCT-116 and SW480 cell proliferation (*n* = 4). **(B)** As determined by colony formation assay, the numbers of colonies in HCT-116 and SW480 were significantly reduced after CASC9 silencing (*n* = 4). **(C)** After knockdown of CASC9, the migration index of HCT-116 and SW480 was significantly increased at both the 24- and 48-h time points (*n* = 6), suggesting decrease in migration of cells to the gap. The data are shown as mean ± SEM compared to the negative control Dsi-NC (*P < 0.05, **P < 0.01).

To evaluate the biological and physiological function of CASC9 in CRC cells, HCT-116 and SW480 cells were silenced by Dsi-CASC9 with Dsi-NC as negative control. By performing CCK-8 assay, we confirmed that CASC9 silencing significantly decreased the cell proliferation capacity in HCT-116 and SW480 cells (Figure 3A). Corresponding to the decrease in cell proliferation, significantly reduced cell growth of HCT-116 and SW480 was shown by Dsi-CASC9 from colony formation assay (Figure 3B). The migration assay was conducted in both cells to evaluate the migration ability of cells. Significant increase of migration index was shown in HCT-116 and SW480 after Dsi-CASC9 treatment (Figure 3C) at 24 and 48 h after transfection.

### Silencing of CASC9-Induced Autophagy in CRC Cells

**FIGURE 4:**
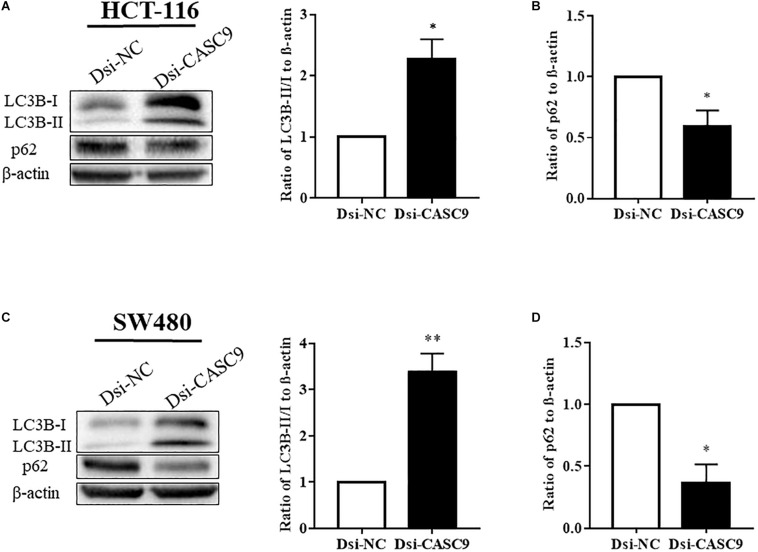
Silencing of CASC9 enhanced autophagy in CRC cells. The expressions of autophagy marker proteins LC3B and p62 were measured by Western blotting in HCT-116 **(A,B)** and SW480 **(C,D)** cells. After Dsi-CASC9–mediated silencing, the ratio of autophagy marker LC3B-II to LC3B-I significantly increased in HCT-116 and SW480 cells with corresponding decrease in p62 expression. The data are shown as mean ± SEM using β-actin as housekeeping control (*P < 0.05, **P < 0.01, and *n* = 4).

Autophagy is a very crucial pathway for cell to survive during energy-deficient and hypoxic conditions. The Western blot in Figures 4A,C showed that the expression of LC3B-II (autophagy marker protein) was significantly upregulated in HCT-116 and SW480 cells after CASC9 silencing. Another autophagy marker protein p62 is a negative regulator of autophagy process. The silencing of CASC9 significantly reduced the expression of p62 protein level in both cell lines (Figures 4B,D), suggesting that Dsi-CASC9 promotes autophagy in HCT-116 and SW480 cells.

### Silencing of CASC9 Promoted the AMPK Signaling Pathway but Downregulated the AKT/mTOR Signaling Pathway in CRC Cells

**FIGURE 5:**
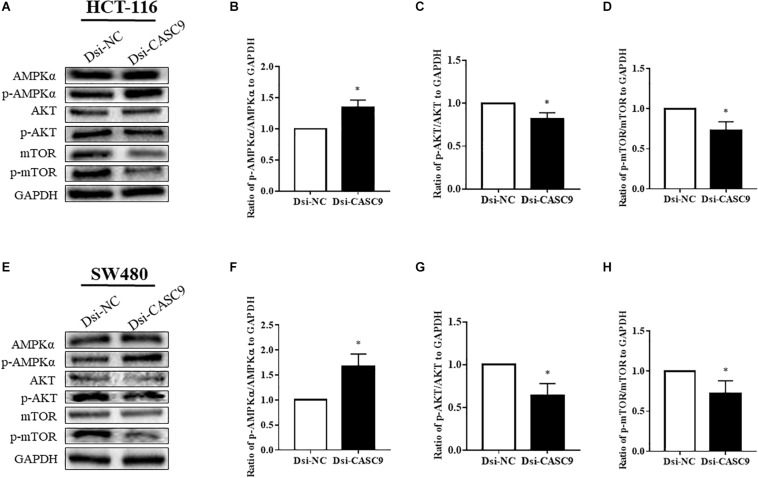
Silencing of CASC9 promoted the AMPK signaling pathway but downregulated the AKT and mTOR pathways. The ratio of p-AMPKα/AMPKα, p-AKT/AKT, and p-mTOR/mTOR to GAPDH were evaluated by Western blotting in HCT-116 **(A–D)** and SW480 cells **(E–H)**. Dsi-CASC9 significantly promotes AMPK signaling in HCT-116 and SW480 cells compared to Dsi-NC. In contrast, CASC9 silencing significantly downregulated AKT and mTOR signaling pathways in HCT-116 and SW480 cells. The data are shown as mean ± SEM using GAPDH as housekeeping gene (*P < 0.05, and *n* = 3).

We subsequently analyze more signaling pathway proteins to explore the role of CASC9 in the AKT and mTOR pathway. The ratio of p-AMPKα/AMPKα, p-AKT/AKT, and p-mTOR/mTOR to GAPDH were being evaluated in HCT-116 (Figures 5A–D) and SW480 cells (Figures 5E–H). Dsi-CASC9 significantly promotes AMPK signaling in HCT-116 and SW480 cells compared to Dsi-NC. In contrast, CASC9 silencing significantly downregulated AKT and mTOR signaling pathways in both cell lines (Figures 5C,D,G,H).

### CASC9 Silencing Altered the Expression of EMT Marker Proteins in CRC Cells

**FIGURE 6:**
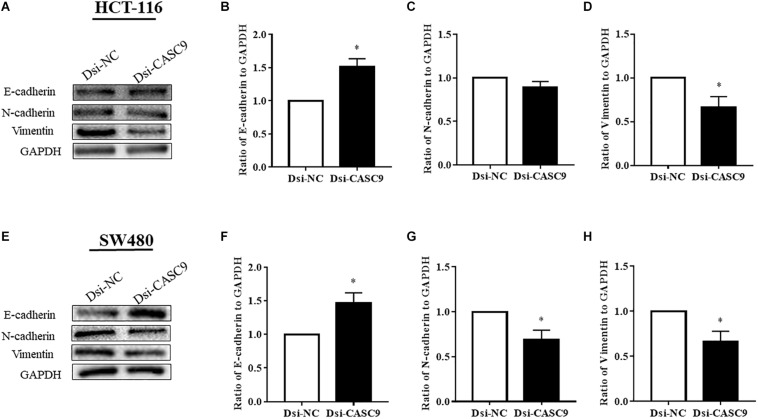
CASC9 silencing altered the expressions of EMT marker proteins in CRC cells. The EMT markers E-cadherin, N-cadherin, and vimentin were evaluated by Western blotting in HCT-116 **(A–D)** and SW480 cells **(E–H)**. In both cell lines, CASC9 significantly upregulated E-cadherin and downregulated vimentin expressions. The N-cadherin expression was also downregulated but did not reach statistical significance in HCT-116 cells. The data are shown as mean ± SEM using GAPDH as housekeeping gene (*P < 0.05, and *n* = 3).

Epithelial–mesenchymal transition is one of the key steps of metastasis in cancer. We explored whether CASC9 silencing would alter the expression of key EMT regulatory proteins, such as E-cadherin, N-cadherin, and vimentin, in HCT-116 and SW480 cells. As shown in Figure 6, the expression of epithelial marker E-cadherin was significantly upregulated in Dsi-CASC9–treated HCT-116 and SW480 cells. On the other hand, the mesenchymal marker protein vimentin was significantly downregulated in HCT-116 and SW480 cells (Figures 6D,H). The N-cadherin expression was also significantly downregulated in SW480 cell (Figure 6G). However, the downregulation of N-cadherin in HCT-116 (Figure 6C) did not reach statistical significance, but the trend was observed in all experiments.

## Discussion

With the advancement of next-generation sequencing technology, an increasing number of lncRNAs have been revealed. LncRNAs are not non-functional by-products or junk molecules of the body (Yao et al., 2019). They play very important roles in epigenetics and have multiple functions in cell growth and development. They are involved in various physiological process related to metabolism including gene mutation, regulation of transcription and translational processes, and regulation of cell cycles (Zhu et al., 2013). In carcinogenesis, lncRNAs play crucial roles in gene expression process steering structural stability and transcriptional process of nucleus, regulating stability of mRNA, and maintaining transcriptional and post-transcriptional modification in the cytoplasm (Wilusz et al., 2009; Marchese et al., 2017; Zampetaki et al., 2018; Yao et al., 2019). So far, approximately 3,000 lncRNAs have been identified from the human genome with their regulatory impact on cancer development, progression, metastasis, and poor prognosis (Gao et al., 2019).

The role of lncRNAs in CRC was first reported by Tsang et al. (2010). They revealed that H19-derived miR-675 plays an oncogenic role in CRC development and progression by targeting retinoblastoma proteins (Tsang et al., 2010). Subsequent studies performed by Kogo et al. (2011) and Xu et al. (2011) demonstrated that HOTAIR and MALAT1 are associated with poor prognosis of CRC by accelerating metastasis process. So far, a large number of lncRNAs have been reported as an oncogenic or tumor-suppressor in CRC such as CDKNIA, PANDAR, MALAT1, CCAT1, CCAT2, UCA1, MEG3, HOTAIR, and GAS5. In addition, ncRuPAR and lincRNA-p21 are considered to be a negative regulator of CRC in the process of radioresistance and metastasis (Zhai et al., 2013; Yan et al., 2014).

In this present study, we first explored the expression of CASC9 in CRC patient samples in two open databases, TCGA-COAD and ENCORI-COAD, which collected cases in the United States and Mainland China, respectively. Even though the plot functions are different in these databases yielding very different graphs (Figure 1), the conclusions are consistent. We observed that CASC9 expression was significantly upregulated in CRC tissues compared to its adjacent normal samples. In addition, the expression of CASC9 is associated with reduced survival of CRC patients. We also demonstrated that CASC9 was overexpressed in CRC cell lines compared to normal colon cell line. All these and previous findings on CASC9 suggested that CASC9 might be a novel marker for CRC prognosis. In fact, there are many reports that CASC9 is involved in non-small cell lung carcinoma, bladder cancer, thyroid cancer, hepatocellular carcinoma, nasopharyngeal cancer, and lung cancer (Jin et al., 2019; Zeng et al., 2019; Chen Y. et al., 2020; Huo et al., 2020; Zhao et al., 2020). It has been suggested that CASC9 is a novel diagnostic, prognostic, and therapeutic target in cancer treatment (Qian et al., 2020; Sharma et al., 2020). Our findings are in line with previous articles in CRC and other cancers (Luo et al., 2019; Ding et al., 2020) and hence leading us to hypothesize that CASC9 may be involved in specific pathogenesis of CRC carcinogenesis.

Many studies have reported that CASC9 knockdown or silencing reduced cell proliferation, invasion, and migration (Jin et al., 2019; Zhang et al., 2019; Chen Y. et al., 2020; Ding et al., 2020; Fang et al., 2020; Huo et al., 2020). We are the first to perform similar experiments in CRC cell lines. By performing a series of *in vitro* experiments, including CCK-8 assay, colony formation assay, and migration assay after Dsi-CASC9 silencing, we confirmed that CASC9 played malignant roles in CRC cell survival, proliferation, and migration. To decipher the role of CASC9 in CRC growth, proliferation, and migration, we examined the potential pathways related to cell growth, apoptosis, and metastasis and decided to focus on autophagy and EMT (Vellai et al., 2008; Wang and Levine, 2010; Sever and Brugge, 2015; Mathiassen et al., 2017; Brabletz et al., 2018; Pavel et al., 2018; Pastushenko and Blanpain, 2019).

The self-degradation mechanism called autophagy is a major intracellular process that maintains the balance between cell death and survival in response to nutritional stress, hypoxia, and growth factor deprivation (Tam et al., 2019; Noguchi et al., 2020). Autophagy is a dual-edged sword that can inhibit or promote carcinogenesis by regulating mTOR and apoptosis process (Levine, 2007). Along with, it is well established that lncRNAs promote or inhibit carcinogenesis by regulating autophagy either through mTOR-dependent or -independent pathways (Peng et al., 2020; Zhang X. Z. et al., 2020). To explore the autophagy process, we determined the expression of autophagy marker proteins LC3B and p62 before and after silencing. Dsi-CASC9 significantly increased LC3B-II and reduced p62 expression. The increased LC3-II is regarded as the standard marker for autophagy. It is directly associated with the number of autophagosomes and considered as the most commonly used autophagic marker protein (Zheng et al., 2012). The ubiquitin-associated protein p62 protein itself is degraded through autophagy and can also serve as a marker of autophagic flux (Cohen-Kaplan et al., 2016; Liu et al., 2016). Here, we clearly demonstrated the promotion of autophagy in CRC after silencing CASC9 (Mizushima, 2004; Jiang and Mizushima, 2015). We believe the induction of autophagy may be related to the reduced cell growth observed after gene silencing.

To further our investigation of molecular pathways in relation with reduced CRC cell proliferation and migration, we explored key signaling molecules, AMPK, mTOR, and AKT, which are linked to the autophagy pathway. In mTOR-dependent autophagy process, AMPK phosphorylates to activate upon energy starvation, leading to phosphorylation of Ser317, Ser777, and Ser555 to activate ULK1 and inhibition of mTORC1 signaling pathway (Paquette et al., 2018; Wang and Zhang, 2019). The protein kinase B or AKT is one of the most critical intracellular pathways associated with mTOR signaling, and it has been considered as the master regulator for most of the cancers (Porta et al., 2014; Yang et al., 2019). Inhibition of AKT/mTOR signaling promotes autophagy and sensitizes tumor cells to anticancer drugs by reducing cell growth, cell cycle, cell survival, differentiation, and metabolism (Paquette et al., 2018; Terracciano et al., 2019). In this study, we revealed that the silencing of CASC9 potentially promotes mTOR-dependent autophagy where it significantly enhances phosphorylation of AMPK and reduces phosphorylation of AKT and mTOR. The inhibition of AKT and mTOR pathways may lead to the attenuated cell growth and migration. Taken together, these findings revealed for the first time that abnormal expression of CASC9 promoted carcinogenesis of CRC cells through activating AKT/mTOR signaling and reduced phosphorylation of AMPK and inhibiting autophagy.

The poor prognosis for most cancer is due to the development of metastasis where EMT eventuates to enhance the cellular migration properties (Brabletz et al., 2018; Wang et al., 2020). In case of CRC, more than 20% of the patients were diagnosed when the tumor has already metastasized to distant organs (van der Geest et al., 2015). Targeted therapy with or without chemotherapy is mostly recommended to patients with advanced stages of CRC for eradication of the tumor (Xie et al., 2020). The traditional targeted or immune therapy mostly targets the abnormal oncogenic proteins or strands of DNAs. Recently, researchers are focusing on using lncRNAs as a novel set of therapeutic targets (Mitra and Chakrabarti, 2018). Our study revealed that CASC9 potentially induced EMT. Here in our evaluation, silencing CASC9 upregulated the epithelial marker protein E-cadherin and downregulated mesenchymal marker protein N-cadherin and vimentin expressions. These results suggest that CASC9 may be involved in CRC progression and metastasis by enhancing the EMT-dependent migratory characteristics of CRC. These findings guide us to propose a novel therapeutic approach that silencing of CASC9 for metastatic CRC patients improves the therapeutic outcomes. However, we still need to wait for the next leap of technology to reach this goal.

## Conclusion

In conclusion, our findings demonstrated that CASC9 was aberrantly upregulated in CRC cells and tissues. Our study revealed that silencing of CASC9 suppressed CRC proliferation, growth, and migration via activation of mTOR-dependent autophagy and inhibition of EMT *in vitro*. This is in line with some recent reports that suggested autophagy/mTOR/EMT pathways may be the crucial targets for the understanding of CRC carcinogenesis and novel therapeutic targets (Song et al., 2019; Wang et al., 2019; Zheng et al., 2019). CASC9 expression in tumor might be a novel prognostic biomarker and CASC9 might be a potential therapeutic target for the management of CRC.

## Data Availability Statement

The datasets presented in this study can be found in online repositories. The names of the repository/repositories and accession number(s) can be found below: https://www.ncbi.nlm.nih.gov/, NR_103850.2, https://www.ncbi.nlm.nih.gov/, NR_103849.2, and https://www.ncbi.nlm.nih.gov/, NR_103848.1.

## Author Contributions

MI and HL conceived and designed the project. MI conducted the experiments, analyzed the data, and wrote the manuscript. HL interpret the results and reviewed the manuscript. Both authors read, approved, and finalized the manuscript.

## Conflict of Interest

The authors declare that the research was conducted in the absence of any commercial or financial relationships that could be construed as a potential conflict of interest.

## Footnotes

1 http://gepia.cancer-pku.cn/detail.php

2 http://gepia.cancer-pku.cn

## Abbreviations

AMPK: AMP-activated protein kinase
ATCC: American Type Culture Collection
CASC9: Cancer susceptibility candidate 9
COAD: Colon adenocarcinoma
CRC: Colorectal cancer
DMEM: Dulbecco modified eagle medium
Dsi-CASC9: Dicer-substrate mediated CASC9
Dsi-NC: Dicer-substrate negative control
EMT: Epithelial–mesenchymal transition
ENCORI: The Encyclopedia of RNA Interactomes
FBS: Fetal bovine serum
HR: Hazard ratio
IDT: Integrated DNA technologies
lncRNAs: Long non-coding RNAs
ncRNAs: Non-coding RNAs
qRT-PCR: Quantitative reverse transcriptase–polymerase chain reaction
TCGA: The Cancer Genome Atlas
TPM: Transcript per million
UNC5B-AS1: The UNC5B antisense lncRNA 1.
